# Self-Organization of Stem Cell Colonies and of Early Mammalian Embryos: Recent Experiments Shed New Light on the Role of Autonomy vs. External Instructions in Basic Body Plan Development

**DOI:** 10.3390/cells5040039

**Published:** 2016-10-25

**Authors:** Hans-Werner Denker

**Affiliations:** Institut für Anatomie, Universität Duisburg-Essen, Universitätsklinikum, Hufelandstr. 55, 45122 Essen, Germany; hans-werner.denker@uni-due.de; Tel.: +49-201-403-792; Fax: +49-201-437-7593

**Keywords:** development, morphogenesis, stem cells, pattern formation, organoids, gastrulation, primitive streak, axis formation, basic body plan, implantation, in vitro models, ethics

## Abstract

“*Organoids*”, i.e., complex structures that can develop when pluripotent or multipotent stem cells are maintained in three-dimensional cultures, have become a new area of interest in stem cell research. Hopes have grown that when focussing experimentally on the mechanisms behind this type of in vitro morphogenesis, research aiming at tissue and organ replacements can be boosted. Processes leading to the formation of organoids in vitro are now often addressed as *self-organization*, a term referring to the formation of complex tissue architecture in groups of cells without depending on specific instruction provided by other cells or tissues. The present article focuses on recent reports using the term self-organization in the context of studies on *embryogenesis*, specifically addressing pattern formation processes in human blastocysts attaching in vitro, or in colonies of pluripotent stem cells (“*gastruloids*”). These morphogenetic processes are of particular interest because, during development in vivo, they lead to basic body plan formation and individuation. Since improved methodologies like those employed by the cited authors became available, early embryonic pattern formation/self-organization appears to evolve now as a research topic of its own. This review discusses concepts concerning the involved mechanisms, focussing on autonomy of basic body plan development vs. dependence on external signals, as possibly provided by implantation in the uterus, and it addresses biological differences between an early mammalian embryo, e.g., a morula, and a cluster of pluripotent stem cells. It is concluded that, apart from being of considerable biological interest, the described type of research needs to be contemplated carefully with regard to ethical implications when performed with human cells.

## 1. Self-Organization: An Emerging Focus of Stem Cell Research

### 1.1. Organoids

An increasing number of publications are dealing with the fact that complex structures, so-called “**organoids**”, can develop when pluripotent or multipotent stem cells (PPSCs and MPSCs) are maintained in three-dimensional cultures in vitro, provided that an appropriate biochemical and physical environment is offered [1,2]. Hopes have grown that when focussing experimentally on these phenomena, research aiming at tissue and organ replacements, or at disease modelling, can find a boost here.

In this context, the term **self-organization** can be read very often in these reports. The term is being used to address processes leading to the formation of complex tissue architecture in groups of cells without depending on specific instructive signals provided by other cells or tissues. Although this term was already known for a long time from classical papers in developmental biology, it was relatively rarely used in the past. That the term is suddenly so much in use in the literature could obviously be for one of two reasons: either our insight into cellular or molecular mechanisms of self-organization has grown to the point that these data can now be discussed on a broad scale, or there is actual reason to intensify asking questions and doing research in this direction. The latter is clearly the case: an arsenal of methods on how to form organoids from stem cells in vitro has been developed (even the industry is offering kits which allow doing this routinely), and investigators are now frequently observing that the complexity of structural order emerging in cultures of PPSCs and MPSCs can be astonishing. Thus, there appears to be an urgent need for systematic work on the cellular and molecular processes going on in these in vitro systems. Consequently, recent initiatives intend to increase research efforts in this direction, like an EMBO conference on organoids being held this year [3] and a Special Issue project of the journal *Development* [4]. In the centre of interest in this field are cell and tissue differentiation, and the formation of spatial order when mimicking organogenesis as closely as possible in three-dimensional systems. Specifically, when including human cells, expectations are high that this type of research will also open new ways for tissue and organ replacement therapies.

### 1.2. “Gastruloids“

In this article I will not deal in detail with such organoids, but will concentrate on the fact that some recent publications are adding another dimension to this type of work when addressing self-organization with regard to **early stages of development**, i.e., **embryogenesis** (in contrast to organogenesis). This type of work deals with stages of development in which processes take place that lead to individuation. This aspect of self-organization appears to be in the process of becoming a research topic of its own, a fact that is just beginning to be recognized by the broader public.

In a report published in 2014, Warmflash et al. [5] described a methodology that allows the manipulation of self-organizational properties of human embryonic stem cells (ESCs) in vitro in such a way that certain of the processes leading to basic body plan (BBP) formation can be mimicked. Specifically, these authors presented a method which applies geometric constraints for manipulating differentiation and pattern formation in ESC colonies and, with appropriate variants of their methodology, they detected an astonishing regularity in the spatial arrangement of the differentiating cells. On one hand, colonies showed pronounced trophoblast differentiation in response to bone morphogenetic protein 4 (BMP4), in agreement with previous work. On the other hand, a remarkable spatial arrangement of these extraembryonic cells, as well as of germ layers, was noted, specifically “*an outer trophectoderm-like ring, an inner ectodermal circle and a ring of mesendoderm expressing primitive-streak markers in between*”. Interestingly, the resulting geometry was dependent on the boundary, i.e., on physical constraints, as demonstrated impressively by culturing on various micro-patterned surfaces. The authors concluded that their observations demonstrate “*an intrinsic tendency of stem cells to make patterns*”, that this “*can be harnessed by controlling colony geometries*”, and that “*geometric confinement … [is] sufficient to trigger self-organized patterning in hESCs*”, under their culturing conditions.

Warmflash et al. interpreted their findings as a proof of principle showing how development of a gastrulating embryonic disc can be engineered in vitro. The resulting colonies which these authors described did not completely mirror the morphology of early postimplantation stage human embryos, however; the outer ring of trophoblast cells, for example, was geometrically different from the trophoblast shell present at the outside of an early postimplantation stage human embryo. Nevertheless, this pile of trophoblast cells could be able to serve functions of localized signal exchange between extraembryonic and intraembryonic cells, as is known to be significant in structuring the embryonic disc and laying down embryonic axes in vivo. On the other hand these engineered embryonic disc-like structures were not elongated or pear-shaped, and the area of cells undergoing epithelial-mesenchymal transition (as in gastrulation) in their culture models was likewise not elongated and not eccentrically located, as a primitive streak (PS) would be in a real embryo. However, those authors expressed their expectation that such differences between the models and real embryos could possibly be corrected in future experiments using improved variants of their technique, specifically other (not round) shapes of a micropatterned matrix/substratum.

Without doubt, interest in their report will be stirred in particular by the fact that these authors [5] expressed the expectation that a wide application of this methodology appears possible, and that it offers a possibility to model processes leading to BBP development in the human, allowing large scale experimentation that could never be feasible (and would have to be considered ethically non-acceptable) with real human embryos. According to what is known from other studies about influences of physical constraints and of extracellular matrices on pattern formation, it may indeed be a realistic perspective that modification of the geometry of the micro-engineered substratum/matrix could lead to the formation of a gastrulation area that comes closer to the morphology of a real PS. Many observations demonstrate that, when early embryonic cells or ESCs are cultivated in vitro, the degree and the kind of order attained strongly depend on physical conditions of culturing, probably by influencing pattern formation/morphogenesis during differentiation of the cells [6,7,8]. Particularly important factors seem to be any extracellular matrix provided as a substratum, and surfaces or other cells to which the stem cells can attach, and their geometric arrangement. In further studies, effects of local sources of morphogens, in combination with micropatterned matrix-coated surfaces, will most probably be used. Thus, it may be expected that the strategies presented by Warmflash et al. [5], combined with engineering approaches tried with non-mammalian vertebrate cells [9], will open avenues of how engineering of a basic body plan (or even embryos) can be pushed towards higher degrees of normality of early in vitro embryonic pattern formation.

Until recently, there had been only a few reports on early embryo-like patterning processes occurring in colonies of pluripotent stem cells. Those earlier reports were not based on systematic studies aimed at elucidating the underlying patterning mechanisms, but they mostly presented findings that had been made incidentally. A particularly impressive example is a report by Thomson et al. [10] on spontaneous formation of a highly structured “embryoid body” in cultures of marmoset monkey ESCs. This remarkably differentiated colony was interpreted by Thomson et al. as an embryonic disc with epiblast and hypoblast, as well as amnion, with an amniotic cavity and a yolk sac. Remarkably, those authors also identified on one end of the epiblast an area which showed all morphological signs of an early PS with its epithelial-mesenchymal transition (i.e., gastrulation). One would have to assume, therefore, that this embryonic disc also seemed to have developed an anterior-posterior (cranio-caudal) axis, thus, an incipient BBP. The morphological similarity to corresponding developmental stages of non-human primates and human embryos is, in fact, astonishing (illustrated and discussed in [11]).

This report by Thomson et al. [10] was, for many years, regarded by most other authors as an overinterpretation of a very exceptional finding. It was only years later that spontaneous pattern formation in stem cell colonies received renewed interest, when gene expression studied in mouse embryoid bodies was found to be unexpectedly similar to patterns typically seen during gastrulation of real mouse embryos in vivo [12]. These authors used reporter constructs in order to visualize Wnt signalling in mouse ESC aggregates and found that “*endogenous Wnt signals polarize the embryoid body and mediate the local execution of a gastrulation-like process*”. This turned out to be a seminal paper stimulating other groups to take a closer look at symmetry breaking processes occurring in embryoid bodies, starting in an apparently autonomous way in suspension culture, or induced (e.g., by attachment to a substratum). For example, Fuchs et al. [13] studied the role of symmetry breaking for cardiomyogenesis in mouse embryoid bodies and found that a timely attachment process promotes breaking of radial symmetry of suspended embryoid bodies.

More recently the term “**gastruloids**” was created to address stem cell-derived structures that exhibited characteristics of gastrulation stages of development [14] (for details of the methodology used see [15]). Those authors observed that small aggregates of mouse ESCs, of about 300 cells, “*self-organised into polarised structures that exhibit collective behaviours reminiscent of those that cells exhibit in early mouse embryos*”. Specifically, in order to stimulate the emergence of PS features, the authors used culture conditions that steer the cells towards this fate, i.e., they exposed embryoid bodies of different sizes to N2B27 medium for two days followed by continuous treatment with both activin A and CHIR99021, a Wnt/β-catenin signalling agonist. As important elements of their protocol, the authors identified the sequence of culture conditions used and the initial number of cells in the aggregate. They found that when applying this protocol “*small aggregates of mESCs undergo a symmetry-breaking event in culture and that, under conditions that promote the formation of mesendoderm in embryos, they exhibit polarised expression of the endoderm marker Sox17 and FoxA2 and of the PS and early mesoderm marker brachyury. Over time, brachyury expression becomes restricted to a small population of cells at a tip of the aggregate, which acts as a source of cells that express Tbx6, a mesoderm gene, and these cells are extruded from the main body of the aggregate in a process that is reminiscent of some of the movements of gastrulation*”. For this reason, they proposed to “*call these aggregates ‘**gastruloids**’ and showed that, although for the most part they are **autonomous** in their development, the culture conditions influence the cell types that develop within them*” (emphasis by HWD). It must be pointed out, however, that in spite of showing these remarkable self-organization capabilities, mouse “gastruloids” were still morphologically less regularly structured as compared to gastrulation stages of embryos developing in utero. A very remarkable aspect of these studies is, in any case, that the symmetry breaking process (i.e., initiation of pattern formation cascades) was found to be able to start autonomously in suspension, i.e., in the absence of observable asymmetry cues acting from the outside. Attachment of the aggregates was found to be not only unnecessary for this pattern formation but disturbing.

The in vitro model of “gastruloids” opens new options for experimental work on cell-to-cell interactions and signalling in mouse embryogenesis. This has already been initiated in the cited study [14]. There is a potential that this type of model studies may provide important additional insight into signalling events involved in axis formation, in shaping of the primitive streak, and in germ layer differentiation, as an important supplement to the gene targeting approach that has been dominating experimental embryology so far (I will come back to this point below when discussing embryos). The emergence of this new branch of stem cell research is remarkable because morphogenesis was originally not seen as a reasonable topic in stem cell research by most authors. It may also help with the recent attempts at defining various levels of potentiality of stem cells more precisely. While a blastocyst has principally full developmental potential, this “totipotency” is usually thought to be lost during derivation of stem cells (ESCs) from the blastocyst, and to be replaced by “pluripotency”, i.e., a reduced level of potentiality excluding an embryo-structuring capability (I will refrain, in this review, from discussing the controversial terminology, e.g., terms like “omnipotency”, “naïve”, and “primed pluripotency”, which has been done before [16]).

Whether a blastocyst possesses specific patterning information for axis formation and positioning/shaping of a PS (which a cluster of PPSCs may be lacking entirely, or be able to develop only to an incomplete degree as long as it does not receive external asymmetry instructions) and how it is encoded is still a matter of debate among embryologists. I will discuss some of the available data further below. Until recently PPSCs were usually considered to have just the potentiality to mimic aspects of organogenesis (in organoids) but not to initiate early embryonic pattern formation processes. Whenever early embryonic pattern formation processes were described in the literature to occur in colonies of ESCs in vitro, such reports were given little credit for many years, and most authors deemed it quite improbable that BBP self-organization potential can be re-gained by ESCs under certain culturing conditions (for a discussion of concepts of developmental biology that do indeed suggest, however, that early embryonic self-organization processes can go on in stem cell colonies under certain conditions; see [11]).

## 2. Self-Organization of Early Embryos

Somewhat surprisingly, two recent publications discuss the terms self-organization and autonomy in the context of the development of early human **embryos** (in contrast to stem cell colonies) in vitro and relate this to blastocyst attachment/implantation [17,18]. One may ask why a morphogenetic potentiality (for self-organization) that had, before, been found astonishing when seen in PPSC colonies, has now become a topic with regard to early embryos. Should this potentiality not be seen as a natural property of blastocysts? That this question is now being discussed has to do with the fact that causal connections have been assumed before to exist between embryo implantation and morphogenesis in mammals.

In the cited investigations, an improved in vitro system was used which had been developed originally for studies in the mouse [19,20]. This culture system has now been applied to study differentiation of donated human embryos (and, partially, also to colonies of human PPSCs: ESCs, and induced pluripotent stem cells, iPSCs). A major feature of the used embryo culture system is that it allows blastocysts to attach to a culture vessel (substratum), thus mimicking one aspect of implantation, but it omits uterine tissues (endometrium) with which the blastocyst would interact during implantation in vivo. In this respect the system is similar to the well-known traditional “embryo outgrowth system”, but it uses improved sequential supplements of growth and attachment factors. The interesting aspect about these papers is that the authors present very detailed studies on the degree of spatial order gained under these conditions in vitro, and they compare the resulting structures with early post-implantation stages of in vivo developed human embryos as known from the Carnegie collection. Although the structures formed in vitro are not really identical with those known from the Carnegie collection embryos, the degree of order attained is, indeed, remarkable as documented in these publications in some detail, morphologically, as well as by applying a panel of germ layer markers. It is in this context that the authors use the term “self-organization”. Specifically, the authors found that these in vitro cultured embryos reached a stage at which their morphology resembled Carnegie stage 5 b–c, with an amniotic cavity, yolk sac cavity, bi-laminar embryonic disc in between, and incipient differentiation of a trophoblast shell. Trophoblast subpopulations self-organized in concentric rings. Some abnormalities were noted, however: At day 12, the amniotic and yolk sac cavities appeared to have collapsed. It is not clear what this would have meant for the survival of these embryos. The authors also noted that “*outgrowths at this late stage lost interpretable relation to* in vivo *correlates…, suggesting the limitations of our two-dimensional culture environment*”. However, the authors obviously considered the possibility that development could have progressed even further; they emphasize that they finished their “*experiments at d.p.f. 14, in accordance with internationally recognized bioethical guidelines*”. They concluded that “*the embryo alone can direct both lineage specification and diversification, as well as tissue morphogenesis and architectural organization, without maternal input*” [17]. In the experiments involving human PPSCs, in which colonies were formed in Matrigel, observed morphogenesis was much more limited, but was interpreted as remarkable, nevertheless: here, cells with epiblast characteristics formed a hollow sphere interpreted as a proamniotic cavity equivalent, but an amniotic epithelium was not formed, possibly because this developmental process requires interaction with extraembryonic tissues (trophoblast, extraembryonic mesenchyme) as present in embryos, but missing here [18]. Clearly, based on the observations made with the explanted blastocysts, the expectation is that morphogenesis could be much more normalized if, in these stem cell cultures, conditions would be chosen that allow for efficient trophoblast formation (which can be stimulated in human PPSC cultures by e.g., BMP).

The reports have immediately received much attention [21,22,23]. Indeed they deserve to be contemplated carefully in at least two respects: (1) with regard to biological theories that have dominated experimental embryology in recent years; and (2) with regard to ethical debates about human embryo research and stem cell derivation and use.

### Autonomy of Early Embryonic Pattern (Basic Body Plan) Formation vs. Dependence on Uterine Instructions

A main point which the cited recent reports are making [17,18] is the notion that, as their results are demonstrating, the formation of spatial order (germ layers and incipient BBP formation) does not depend on implantation of a human blastocyst in the uterus, since their in vitro system does not include uterine tissues (endometrium), and that a remarkable degree of structural order can be reached, nevertheless. Upon comparing with the known Carnegie stages of human development in vivo, the authors conclude that these in vitro attached embryos have reached a state of morphogenesis that could have allowed them to let BBP development continue during subsequent gastrulation/primitive streak (PS) formation. However, in order to conform to internationally accepted ethical rules the authors have stopped culturing these embryos for longer time periods in order to prevent them from forming a PS. The authors as well as the commentators [21,22] emphasize as a remarkable new information the fact that the embryos reached a relatively advanced stage of development in vitro.

Indeed, surprisingly many people have, for a long time, favoured (and some still seem to be favouring) the view that implantation in the uterus is instrumental in providing axis-determining signals necessary for BBP formation. This assumption ascribing such a decisive morphogenetic role to implantation was, however, based almost entirely on observations and experiments performed in the mouse, not in other species. The assumption was upheld even though conflicting observations had been made by some authors who showed that asymmetries anticipating the a-p axis can develop autonomously, in the mouse ([24,25]). This is in good agreement with old work by Hsu et al. on in vitro development of mouse embryos to very advanced stages [26,27]. When considering what is known from comparative embryology it must appear strange, however, that the idea about an instructive role of embryo implantation was so much favoured (and even generalized to include other species, like humans) by many authors until recently. A comparative view at embryo implantation shows clearly that extrapolation from the mouse to other species is difficult and must be done with caution. In the first place it must not be forgotten that in many species, axis/BBP formation starts well before implantation. This is typically seen in those species which represent the central mode of implantation (where the conceptus remains topographically in the former uterine lumen, notwithstanding the fact that the intimacy of the cellular contact formed between the trophoblast and endometrial tissues during placentation differs between species, and that it can even be hemochorial, like in rabbits). For example, in horses, the BBP of the embryonic anlage is already far advanced at the time when the conceptus is still non-implanted but contained within its coats (“capsule”), and it is only many days later that the trophoblast starts attaching to, and invading into, the endometrium [28]. In rabbits the anterior-posterior polarity of the embryonic disc is established already before hatching from the blastocyst coats/coverings, and the primitive streak begins to form before attachment to the endometrium starts [29,30,31].

**Figure 1:**
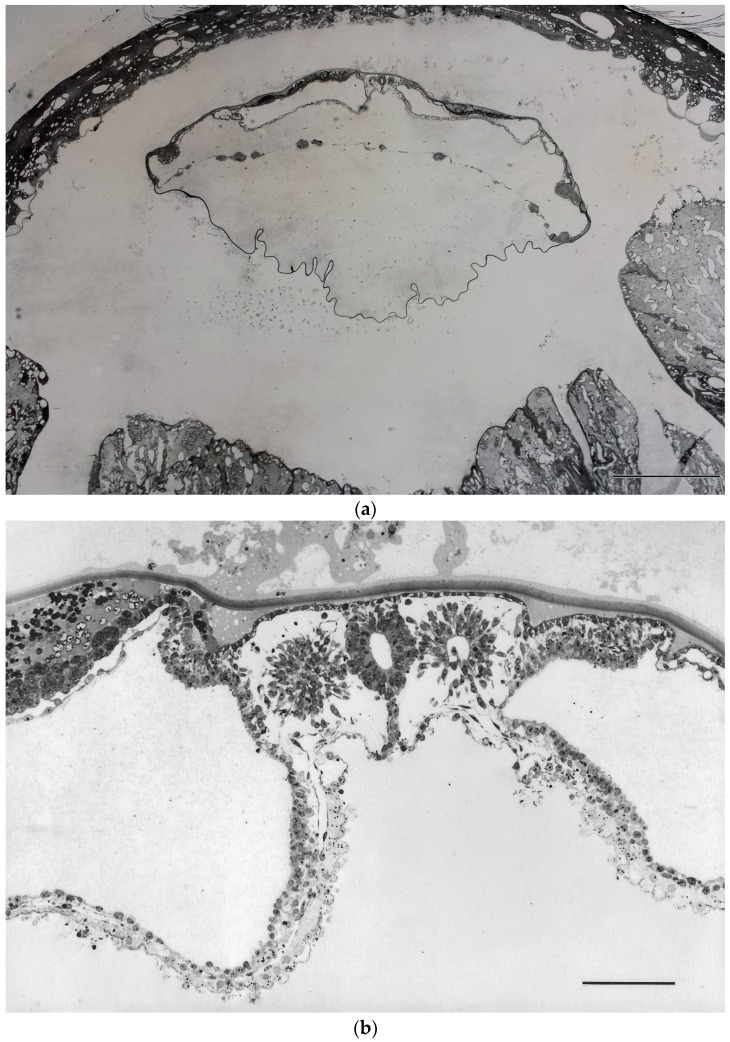
Basic body plan development has been shown to be independent of implantation in the uterus in vivo in rabbits. In these experiments, hatching of the blastocyst from its coats/coverings (zona pellucida equivalents) was blocked by continuous intrauterine infusion of a non-toxic proteinase inhibitor, aprotinin. As a result, the embryo shown here is still completely encased in its glycoprotein coats, and the trophoblast has not been able to attach to the endometrium and start its hemochorial placentation. Nevertheless, early embryonic pattern formation processes (basic body plan) have not only set in, but have continued to a remarkable degree, thus demonstrating independence of implantation. The stage shown is 9 ½ days post coitum, i.e., 2 ½ days after implantation has started in the controls. Spaces between the surfaces of the conceptus and the endometrium are exaggerated due to shrinkage during dehydration and Araldite embedding. (**a**) Overview; bar = 1 mm; (**b**) higher magnification of anlage of embryo proper; bar = 100 µm. Neural tube, notochord, somites, somatopleure and splanchnopleure etc. show normal morphology. Reproduced with permission, from [33] (a,b); [34] (a).

Independence of BBP formation from implantation had indeed not only been suggested by such morphological findings, but it has also been demonstrated experimentally in vivo, in the rabbit. These experiments, performed already quite some time ago [32,33,34], have shown that BBP development does not only start, but can progress, quite normally when blastocyst implantation is inhibited in vivo, using non-toxic inhibitors of the key proteinase system involved in hatching: in these experiments blastocysts were found to remain enclosed in their extracellular coverings (coats) so that they were unable to establish a cellular contact to the uterine tissues. Still, they continued developing, forming axes, neural tube, somites, etc., i.e., a stage of development well beyond the primitive streak stage, although they were still free-floating in the uterine lumen (Figure 1) [33,34].

Why then was the literature dominated, until recently, by the notion that essential instructory signals for axis determination are to be provided by implantation in the uterus, so that the findings presented in the recent papers [17,18] were considered so remarkable by commentators? This has a peculiar history. Years ago a correlation had been found between the orientation of axes of the developing mouse embryo and of the uterus [35,36,37,38]. Although a correlation does not demonstrate an underlying mechanism, and although several mechanisms can be imagined to cause the described regularity (e.g., spatially oriented implantation), it was assumed that the uterus provides instructory, axis-determining signals needed by the blastocyst, and this assumption became, for many years, like a dogma. It was no earlier than around 1990 that some of the experimental embryologists working in the mouse system started questioning this concept. A good illustration is the following citation from a paper by Richard Gardner [39]: “*Radial symmetry around [the] embryonic-abembryonic (EM-AB) axis is generally assumed to persist through implantation until the beginning of gastrulation when the appearance of the primitive streak (PS) serves to define the posterior of the future fetus. According to this view, the only fetal axis that can be assigned before gastrulation is the dorso-ventral one because it happens to correspond to the EM-AB axis of the blastocyst and egg-cylinder stage*” (for a more recent review which reflects a state of discussion reached when dominant views about mechanisms were already in a process of changing, see [37]). The dogma that development of definitive body axes of the embryo is strictly dependent on instructions from the uterus, although originally developed with regard to mouse development, played a significant role even in discussions about the ethics of human embryo research and stem cell derivation, at least in Germany (in the early 2000s): It was argued that the time point for the beginning of individual (organismic) life cannot reasonably be set at zygote formation but at a post-zygotic stage, specifically at implantation, since, according to this concept, to develop a capacity for BBP formation would require information provided by embryo implantation in the uterus. This was regarded by politicians as an important argument to ask for liberalization of the use of preimplantation embryos for stem cell derivation. Findings like those addressed above about BBP formation in embryos that had been experimentally prevented from implanting (as discussed above, Figure 1), were simply disregarded in those argumentations.

Unfortunately, the mouse is far from being an ideal model for human embryology, as well as implantation, for a number of reasons: On one hand, the endocrinology of implantation, the mode of decidualization and the cell biology of the uterine epithelium differ considerably from the situation in many other species, including humans. In the context discussed here it is particularly relevant that the topography of germ layer stages in mice cannot be easily compared with that of other species. Early post-implantation stages of the mouse present a so-called germ layer inversion (the egg cylinder). Comparing with the great majority of species (including humans) which do not have a germ layer inversion, but rather a flat embryonic disc, is hampered by this fact. Embryologists use, therefore, theoretically-deduced flattened maps of mouse egg cylinders in order to facilitate comparison [40]. Moreover, it cannot be excluded that signalling events relevant for axis and BBP formation may differ in detail between the mouse egg cylinder and the naturally flat embryonic disc of other species, since distances which must be breached (by transport/diffusion of signalling molecules) are necessarily quite different in these two topographical situations (discussed in [41]).

Most of the investigations on involved genes and on molecular signalling events have, nevertheless, been performed in the mouse, due to its amenability to studies involving genetic markers and gene targeting. A peculiarity of the mouse is, as mentioned above, that the anlage of the embryo proper appears to be, in the egg cylinder stage, at first radially symmetrical, without any anterior-posterior (a-p) axis being detectable morphologically. The concept favoured by most authors implies that a process of **symmetry breaking** occurs subsequently when an a-p axis develops, apparently de novo, and becomes superimposed on the proximo-distal axis of the egg cylinder. Based on this concept quite a number of detailed investigations have been performed, in mice, with the aim to uncover molecular details of gene expression and of signalling events involved in this process of symmetry breaking, as well as of cell movements. Of particular importance for the firm establishment of the a-p axis is the signalling centre of the anterior visceral endoderm (AVE) [25,40,42]. The exact origin of the instructions that are instrumental in transforming the radial symmetry of the egg cylinder into an a-p asymmetry/axis still remains largely obscure, however. Does the pre-implantation blastocyst already have such information, perhaps in a cryptic form? Does it need to receive structuring information from an external source, i.e., the uterus?

A re-focussing started when, using gene expression markers, evidence was found that the orientation of the a-p axis is already anticipated before AVE migration [24,43]. However, it remained unclear whether e.g., the tilt of the embryoblast that is typically observed in mouse blastocysts, or even earlier asymmetries in cleavage stages and blastocysts (i.e., clearly before implantation) [38,44,45], may play a role in providing cues that are relevant for a-p axis development. There is evidence that cross-talk between cells of the extraembryonic membranes (like the AVE mentioned above, but also the trophoblast) on one hand, and cells of the embryo proper (primitive ectoderm/epiblast) on the other, are crucial in axis determination (for a review see [25]). Since the trophoblast forms the outer layer of the conceptus that is in direct contact with the endometrium during implantation in vivo, it did not appear unreasonable to think of the possibility that any asymmetry in the interaction of the trophoblast with endometrial tissues at implantation might be transmitted to the embryo proper, thus providing instructions for axis positioning and patterning. Experiments showing this positively are lacking, however, as is any information on the nature of such possible signals.

The recent research on “gastruloids” formed by ESC clusters in vitro, without attachment and without contact to uterine tissues [14], discussed above, appears to not only provide new experimental access to studying these processes but also to shed new light on signalling processes in mouse embryos as taking place in vivo, and it may modify some of the views so far prevailing. For example van den Brink et al. [14] summarize the concepts about signalling at axis and PS formation, and new aspects emerging from their in vitro work on “gastruloids”, as follows (references omitted): The “*initial localisation of the PS can be identified as a focus of Bra expression in the proximal posterior region of the embryo …, and its specification follows a sequence of events associated with the localisation of ligands for BMP, Nodal and Wnt signalling to the same region … This process requires first the specification and localisation of the anterior visceral endoderm (AVE) to the prospective anterior region of the conceptus, where it acts as a source of antagonists of Wnt, BMP and Nodal signalling … It is thought that the action of the AVE positions or restricts the PS to the opposite end of the epiblast … Our results [as obtained with “gastruloids” in vitro; HWD] raise questions about the actual role of the AVE, since they show that a stable axis, as reflected by localised expression of Bra, Sox17 and FoxA2, can be initiated **without external influences** [emphasis by HWD]. In our experiments the signals are ubiquitous and so **the symmetry-breaking event must be intrinsic to the aggregates, raising the possibility that a similar spontaneous event takes place in the embryo** [emphasis by HWD]. This conclusion is at odds with the large body of experimental evidence suggesting that the antero-posterior axis requires a sequence of interactions between extraembryonic and embryonic tissues … One way to reconcile our observations with those of the genetic analysis of early development would be to entertain the possibility that the function of the AVE is not to break the symmetry of the embryo but rather to ensure that an event that can happen spontaneously has a reproducible outcome …*”

On the basis of these findings, as well as of the experiments on inhibition of implantation by proteinase inhibitors in vivo in rabbits (see above, Figure 1), the observations presented in the abovementioned recent publications that the development of spatial order in human embryos in vitro can occur independent of implantation in the uterus [17,18], should, therefore, not really be considered too surprising. The fact that commentators found it so remarkable can probably be understood best in the light of the outlined history that was dominated by gene targeting experiments performed in the mouse and is now receiving supplemental aspects from the mentioned in vitro model studies.

## 3. The Biological Role of Gastrulation in BBP Formation, and Ethical Implications

Culturing human embryos in vitro in peri- and post-implantation stages of development raises ethical and legal questions. This is obviously one reason why a number of comments already appeared simultaneously with the cited recent papers on human blastocyst self-organization in vitro [17,18]. In these comments [21,22,23] the question was raised whether the demonstrated feasibility of letting differentiation of human blastocysts progress to reach the equivalents of early postimplantation stages in a simple in vitro system should be seen as an argument for lifting now certain legal restrictions on experimentation with early human embryos, since this clearly invites continuing investigations by others using these model systems. So far, rules established in many countries [23] have set a limit for experimentation with early human embryos at an embryo age of 14 days or the primitive streak (PS) stage, whichever comes first. This is based on the recommendations of the Warnock Commission (an interesting account of the history of these recommendations and their forging into legislation in the UK can be read in [46]). To choose the 14 day/PS stage as a legally relevant limit was anything but arbitrary, but was the result of extensive counselling and discussions in the UK Parliament centring on the question “When does life begin?”. Mary Warnock summarized the main aspects dominating these discussions as follows: “*What we were really asking was ‘When, in the gradual development of the embryo do we begin to think of it as something that merits protection? What, at its various stages, is to be its moral status?’* ” [46]. The result of these discussions was to define as a limit the 14 day/PS (gastrulation) stage. In recent years, an aphorism by Lewis Wolpert has become very popular, accentuating the biological view behind these regulations, and it has been repeated in countless publications (including the preface of Claudio Stern’s excellent book on gastrulation [47]): “*The most important event in our life is not birth, marriage, or death but gastrulation*”. Should there now be reason to suddenly deviate from putting so much emphasis on the ethical implications of gastrulation/PS formation and to liberalize, from now on, research including these stages of development?

In the following paragraphs I will give a brief overview over the facts which developmental biology tells us with regard to gastrulation/PS formation, and their relevance for BBP formation. This does not mean that I subscribe to Wolpert’s dictum (I do see development as a continuum after zygote formation). Ordered gastrulation as it occurs in the PS is of central importance for the formation of a BBP (reviewed by [11,47]). The PS is instrumental in making sure that a normal body plan with its anterior-posterior axis is laid down, that a singleton is formed (in contrast to monozygotic twins), and that the result is not a chaotic mixture of tissues (teratoma). The anterior part of the PS (Hensen’s node) acts as an organizer, comparable to the Spemann-Mangold organizer in amphibian embryos. The application of molecular approaches to developmental biology has, in recent years, led to an impressive gain in knowledge about the mode of action of the organizer, its origin, and its functional significance during the formation of the BBP. Without an organizer the embryo does not develop an orthotopic and normally-structured notochord, branchial apparatus, central nervous system, and post-anal tail. The products of appropriate deletion experiments are similar to teratomas (for references, see [11]). Doubling of the organizer and the PS leads to the formation of monozygotic twins (conjoint twins in the case of incomplete separation of parts of the bodies). Thus, the PS and its organizer are of central importance for individuation.

The organizer is, itself, induced in amphibia by the Nieuwkoop centre, whose position defines the future body axes. The organizer is formed during a series of hierarchically-arranged vectorial events that, at many points, allow for regulative processes. First axis information is apparently derived from very simple asymmetries, starting in amphibia with cytoplasmic asymmetries of the oocyte, modified by the penetration of the sperm (in the chick by gravitation and egg rotation). Some authors have presented data suggesting that this mode of generating the first asymmetries, defined by the site of sperm entry into the oocyte, may also be in use in the mammalian system [44]. Controversial views have been presented on the question where the information originates which first establishes the relatively simple asymmetries needed, which are thereafter elaborated to form embryonic axes as just described: asymmetries (1) of the oocyte; (2) of the zona pellucida; (3) of the zygote, after reorganization of the oocyte cytoplasm as triggered by sperm penetration; (4) positioning of the pronuclei in the zygote; (5) stochastic positioning of the inner cell mass occurring at blastocyst cavity formation; or (6) any asymmetries induced somehow during interaction with the endometrium at implantation (as discussed above) ([11,25,36,38,44,45]). Whatever the nature of the first meaningful asymmetries may be, this information must be translated into the formation of the BBP in a cascade of processes (cytoplasmic movements, segregation, proliferation, cell movements, induction processes). Depending on the concept (1–6), either the oocyte/zygote, or one of the subsequent stages of preimplantation development, or an early implantation stage, must be assumed to possess all the information necessary to run this developmental program. For the investigator, the situation is complicated by the fact that, as experimental evidence shows, axis information is first laid down in the form of a pre-pattern that in the beginning is not rigid but still allows for some modification. The system leaves room for regulative processes on many levels as long as the patterns have not yet been specified in every detail (discussed in [11]).

The recent high interest in self-organization of stem cell colonies mentioned in the introduction must raise the question whether processes leading to formation of an organizer and PS can also take place spontaneously in colonies of PPSCs in vitro. Reasons why this does not appear improbable have been discussed before [11]. As suggested by computer modelling (Turing model) [48,49], stochastically arising heterogeneities occurring in vitro may suffice as surrogate asymmetry centres in PPSC colonies, in the absence of (e.g.,) zygote-derived (or uterus-derived) axis-determining signals. As long as it depends on such stochastic asymmetries, the development of complex morphogenetic processes in vitro can be expected to be an infrequent event, but these processes can probably be engineered to go on more regularly and to reach BBP patterns that come closer to those seen in normal embryos, when strategies are applied as described in some of the recent studies [5]. If (as discussed above) interactions between extraembryonic (trophoblast) and embryoblast-type cells play a role in this context, even this interaction can quite probably take place in PPSC colonies: the occurrence of trophoblast in human PPSC cultures under certain conditions has, in the meantime, been corroborated in many publications (for a literature list see [50]) (although the low tendency of trophoblast differentiation in mouse, in contrast to primate, ESC cultures had often been used as an argument to the contrary in the past; a fact that has led to confusions in discussions about the ethics of stem cell work).

Finally, recent reports on so-called 2C-like cells (2CLCs) are of particular interest in this context. These 2CLCs may, indeed, provide an additional tool for experimental investigation of some of the questions just discussed about the self-organization potential. 2CLCs occur spontaneously in PPSC cultures in low frequency as a subpopulation [51]. Methods have now been described which allow to increase the percentage of such cells in the cultures substantially by epigenetic manipulation [52]. In the cited studies, the authors depleted either the p150 or the p60 subunits of chromatin assembly factor-1 (CAF-1) in mouse ESCs. This led to increased accessibility at MERVL and to up-regulation of neighbouring genes, thus generating a transcriptional profile similar to that of two-cell-stage embryos. The authors reported that the 2CLC stem cells resembled blastomeres isolated from two-cell stage embryos not only with regard to gene expression patterns, but also to the capacity to reactivate transcription of endogenous retroviruses, as well as to the embryo-forming capacity gained during reprogramming by nuclear transfer to oocyte cytoplasm. Both blastomeres of two-cell stage embryos are known to be totipotent in the strict sense, i.e., capable of performing complete development in vivo. Thus, the question arises whether these 2CLC stem cells might express self-organization capabilities that exceed those known from traditional ESC colonies, if tested in appropriate experimental settings (e.g., in an empty zona, or in the setting described by [5]). 2CLCs are much smaller than two-cell blastomeres, and they cannot be expected to possess any axis-preinformation provided via cytoplasmic determinants derived from the zygote (as postulated by one of the existing theories, mentioned above). As we have discussed, self-organized early embryonic pattern formation can apparently be stimulated in colonies of stem cells by surrogate signals, e.g., asymmetries in cell densities or physical constraints, as well as the structure of the extracellular matrix. If the potential of 2CLCs would be tested by, e.g., transferring the cells into an empty zona (providing a neutral environment excluding preimplantation asymmetry signals), it could be seen whether autonomous morphogenesis would be possible or impossible in this case. Effects of the addition of an artificial local source of morphogen could be investigated. Such experiments could shed light on the question what exactly the difference might be between a (totipotent) morula and a cluster of PPSCs, in particular of 2CLCs, which is still a conundrum at this moment. Taking as an example a somewhat later stage, the same can be said about the nature of differences between a blastocyst and an engineered trophoblastic vesicle with an inside cluster of PPSCs, e.g., 2CLCs (experiments that are a variant of the chimera formation and tetraploid complementation assays). In order to determine how close the biological properties of 2CLCs may come to those of two-cell blastomeres, it could appear interesting to study in such experiments whether or not a regular PS (and, thus, an incipient BBP) can be formed autonomously in vitro. However, such experiments should not be performed in the human but with non-human primate PPSCs, in order to avoid any formation of a human BBP in vitro [16,53].

In conclusion, this review has focussed on autonomy vs. heteronomy of BBP formation, because it appears to me that this question has implications for the ethics of working with human embryos and stem cells. Some argue that it is impossible to derive any kind of ethics from biological fact. While this may generally be correct, aspects of organismic wholeness and of autonomy have been, and are, playing a role in discussions about ethical questions, concerning the end of life (clinical death and organ donation), as well as the beginning of life (Warnock report and UK legislation, addressed above). Arguments along these lines are often not without inconsistencies and contradictions. For example, the argument that post-PS stage embryos should deserve a higher degree of protection due to the fact that from that stage on (complete) twinning is not possible anymore, used as an indication of achieved individuation, is obviously in conflict with using potentiality as an argument for deserving protection: it might be argued that the end of twin formation capability indicates a loss of a remarkable developmental potentiality. In order to avoid ambiguities of the potentiality argument, it appears helpful to focus on autonomy and the gain of organismic wholeness as relevant aspects, in the discussion of ethical implications. Biological facts reviewed in the preceding paragraphs thus concentrate on aspects of autonomy and organismic wholeness.

Development is a continuum from the zygote stage on, and any quantum leaps that one may like to detect here, are artificial. As we have learned from the recent data, embryo development is, in terms of morphogenetic mechanisms, not dependent on outside instructions (e.g., from the uterus). With regard to the ethics of experimentation with human embryos and PPSCs (cf. the cited observations on self-organization of human blastocysts in vitro [17,18], as well as the studies on stem cell colony differentiation [11,16]), I cannot see that any arguments can be derived here which would justify abandoning the 14 day (PS stage) limit for experimentation, as argued for in some comments [22,23]. The recent findings discussed in the present review have shown a higher degree of autonomy (capability for self-organization) than expected before, in PPSC colonies as well as in blastocysts. If it is intended to draw any conclusions of ethical relevance from these new findings, I think this can logically not be a call for less protection. A main argument in the gradualistic concepts for ethical regulations concerning embryo research is that the stepwise gain of increasing grades of organismic wholeness and autonomy is a crucial aspect of individuation, no matter at what point in development one would perhaps like to see a quantum leap and to use this as a criterion sufficient for having reached a new dignity (e.g., fertilization, implantation, neurulation, or any of the levels of brain function development). More autonomy must, according to such logic, equal to more, not less dignity. I, thus, argue that these new findings can, logically, only ask for granting more, not less, protection to the earliest (preimplantation) stages of human development, and for more stringent restrictions concerning self-organization experiments with human PPSCs.

## Abbreviations

AVE: anterior visceral endoderm; a-p: anterior-posterior; BBP: basic body plan; ESC: embryonic stem cell; iPSC: induced pluripotent stem cell; MPSC: multipotent stem cell; PPSC: pluripotent stem cell; PS: primitive streak; 2CLC: 2-cell-like cell.

## References

[1] Willyard C. (2015). Rise of the Organoids. Biologists are building banks of mini-organs, and learning a lot about human development on the way. Nature.

[2] Clevers H. (2016). Modeling Development and Disease with Organoids. Cell.

[3] EMBO/EMBL Symposium ‘Organoids: Modelling Organ Development and Disease in 3D Culture’, Heidelberg (Germany), 12–15 October 2016. http://www.embo-embl-symposia.org/symposia/2016/EES16-07/programme1/index.html.

[4] Development (The Company of Biologists; ed.), Special Issue on Organoids (to be published 2017). http://dev.biologists.org/content/special-issue-organoids.

[5] Warmflash A., Sorre B., Etoc F., Siggia E.D., Brivanlou A.H. (2014). A method to recapitulate early embryonic spatial patterning in human embryonic stem cells. Nat. Methods.

[6] Behr R., Heneweer C., Viebahn C., Denker H.-W., Thie M. (2005). Epithelial-mesenchymal transition in colonies of rhesus monkey embryonic stem cells: A model for processes involved in gastrulation. Stem Cells.

[7] Maranca-Hüwel B., Denker H.-W. (2010). Epithelial-mesenchymal transition in rhesus monkey embryonic stem cell colonies: The role of culturing conditions. In Vitro Cell. Dev. Biol. Anim..

[8] Poh Y.C., Chen J., Hong Y., Yi H., Zhang S., Chen J., Wu D.C., Wang L., Jia Q., Singh R. (2014). Generation of organized germ layers from a single mouse embryonic stem cell. Nat. Commun..

[9] Xu P.F., Houssin N., Ferri-Lagneau K.F., Thisse B., Thisse C. (2014). Construction of a vertebrate embryo from two opposing morphogen gradients. Science.

[10] Thomson J.A., Kalishman J., Golos T.G., Durning M., Harris C.P., Hearn J.P. (1996). Pluripotent Cell Lines Derived from Common Marmoset (Callithrix jacchus) Blastocysts. Biol. Reprod..

[11] Denker H.-W. (2004). Early human development: New data raise important embryological and ethical questions relevant for stem cell research. Naturwissenschaften.

[12] Ten Berge D., Koole W., Fuerer C., Fish M., Eroglu E., Nusse R. (2008). Wnt signaling mediates self-organization and axis formation in embryoid bodies. Cell Stem Cell.

[13] Fuchs C., Scheinast M., Pasteiner W., Lagger S., Hofner M., Hoellrigl A., Schultheis M., Weitzer G. (2012). Self-Organization Phenomena in Embryonic Stem Cell-Derived Embryoid Bodies: Axis Formation and Breaking of Symmetry during Cardiomyogenesis. Cells Tissues Organs.

[14] Van den Brink S.C., Baillie-Johnson P., Balayo T., Hadjantonakis A.K., Nowotschin S., Turner D.A., Martinez Arias A. (2014). Symmetry breaking, germ layer specification and axial organisation in aggregates of mouse embryonic stem cells. Development.

[15] Baillie-Johnson P., van den Brink S.C., Balayo T., Turner D.A., Arias A.M. (2015). Generation of Aggregates of Mouse Embryonic Stem Cells that Show Symmetry Breaking, Polarization and Emergent Collective Behaviour in Vitro. J. Vis. Exp..

[16] Denker H.-W. (2014). Stem cell terminology and ‘synthetic’ embryos: A new debate on totipotency, omnipotency, and pluripotency and how it relates to recent experimental data. Cells Tissues Organs.

[17] Deglincerti A., Croft G.F., Pietila L.N., Zernicka-Goetz M., Siggia E.D., Brivanlou A.H. (2016). Self-organization of the in vitro attached human embryo. Nature.

[18] Shahbazi M.N., Jedrusik A., Vuoristo S., Recher G., Hupalowska A., Bolton V., Fogarty N.M., Campbell A., Devito L.G., Ilic D. (2016). Self-organization of the human embryo in the absence of maternal tissues. Nat. Cell Biol..

[19] Bedzhov I., Leung C.Y., Bialecka M., Zernicka-Goetz M. (2014). In vitro culture of mouse blastocysts beyond the implantation stages. Nat. Protoc..

[20] Bedzhov I., Zernicka-Goetz M. (2014). Self-Organizing Properties of Mouse Pluripotent Cells Initiate Morphogenesis upon Implantation. Cell.

[21] Rossant J. (2016). Human embryology: Implantation barrier overcome. Nature.

[22] Reardon S. (2016). Human embryos grown in lab for longest time ever. Embryos cultured for up to 13 days after fertilization open a window into early development. Nature.

[23] Hyun I., Wilkerson A., Johnston J. (2016). Embryology policy: Revisit the 14-day rule. Nature.

[24] Takaoka K., Yamamoto M., Hamada H. (2011). Origin and role of distal visceral endoderm, a group of cells that determines anterior-posterior polarity of the mouse embryo. Nat. Cell Biol..

[25] Takaoka K., Hamada H. (2012). Cell fate decisions and axis determination in the early mouse embryo. Development.

[26] Hsu Y.C. (1979). In vitro development of individually cultured whole mouse embryos from blastocyst to early somite stage. Dev. Biol..

[27] Chen L.T., Hsu Y.C. (1982). Development of mouse embryos in vitro: Preimplantation to the limb bud stage. Science.

[28] Betteridge K.J., Eaglesome M.D., Mitchell D., Flood P.F., Beriault R. (1982). Development of horse embryos up to twenty two days after ovulation: Observations on fresh specimens. J Anat..

[29] Idkowiak J., Weisheit G., Viebahn C. (2004). Polarity in the rabbit embryo. Semin. Cell Dev. Biol..

[30] Viebahn C., Mayer B., de Angelis M.H. (1995). Signs of the principle body axes prior to primitive streak formation in the rabbit embryo. Anat. Embryol..

[31] Viebahn C., Stortz C., Mitchell S.A., Blum M. (2002). Low proliferative and high migratory activity in the area of Brachyury expressing mesoderm progenitor cells in the gastrulating rabbit embryo. Development.

[32] Denker H.-W. (1977). Implantation. The role of proteinases, and blockage of implantation by proteinase inhibitors. Adv. Anat. Embryol. Cell Biol..

[33] Denker H.-W., Meinshausen E. Continued differentiation of mammalian embryos in utero after blockage of implantation. Proceedings of the XVth EDBO International Embryological Conference (EDBO).

[34] Meinshausen E., Denker H.-W. (1983). Entwicklungsleistungen von Kaninchenembryonen trotz Hemmung der Anheftung in utero. Verh. Anat. Ges..

[35] Smith L.J. (1980). Embryonic axis orientation in the mouse and its correlation with blastocyst relationships to the uterus. Part 1. Relationships between 82 hours and 4 1/4 days. J. Embryol. Exp. Morphol..

[36] Gardner R.L. (2001). Specification of embryonic axes begins before cleavage in normal mouse development. Development.

[37] Rossant J., Tam P.P.L. (2004). Emerging asymmetry and embryonic patterning in early mouse development. Dev. Cell.

[38] Gardner R.L. (2006). The axis of polarity of the mouse blastocyst is specified before blastulation and independently of the zona pellucida. Hum. Reprod..

[39] Gardner R.L., Meredith M.R., Altman D.G. (1992). Is the anterior-posterior axis of the fetus specified before implantation in the mouse?. J. Exp. Zool..

[40] Tam P.P.L., Gad J.M., Stern C. (2004). Gastrulation in the mouse embryo. Gastrulation: From Cells to Embryo.

[41] Fischer B., Chavatte-Palmer P., Viebahn C., Navarrete Santos A., Duranthon V. (2012). Rabbit as a reproductive model for human health. Reproduction.

[42] Rossant J., Tam P.P. (2009). Blastocyst lineage formation, early embryonic asymmetries and axis patterning in the mouse. Development.

[43] Torres-Padilla M.E., Richardson L., Kolasinska P., Meilhac S.M., Luetke-Eversloh M.V., Zernicka-Goetz M. (2007). The anterior visceral endoderm of the mouse embryo is established from both preimplantation precursor cells and by de novo gene expression after implantation. Dev. Biol..

[44] Piotrowska K., Zernicka-Goetz M. (2001). Role for sperm in spatial patterning of the early mouse embryo. Nature.

[45] Motosugi N., Bauer T., Polanski Z., Solter D., Hiiragi T. (2005). Polarity of the mouse embryo is established at blastocyst and is not prepatterned. Genes Dev..

[46] Warnock M. (2001). Anne McLaren as teacher. Int. J. Dev. Biol..

[47] Stern C.D. (2004). Gastrulation—From Cells to Embryo.

[48] Meinhardt H. (2006). Primary body axes of vertebrates: Generation of a near-Cartesian coordinate system and the role of Spemann-type organizer. Dev. Dyn..

[49] Meinhardt H. (2012). Turing’s theory of morphogenesis of 1952 and the subsequent discovery of the crucial role of local self-enhancement and long-range inhibition. Interface Focus.

[50] Denker H.-W. (2012). Time to reconsider stem cell induction strategies. Cells.

[51] Macfarlan T.S., Gifford W.D., Driscoll S., Lettieri K., Rowe H.M., Bonanomi D., Firth A., Singer O., Trono D., Pfaff S.L. (2012). Embryonic stem cell potency fluctuates with endogenous retrovirus activity. Nature.

[52] Ishiuchi T., Enriquez-Gasca R., Mizutani E., Bošković A., Ziegler-Birling C., Rodriguez-Terrones D., Wakayama T., Vaquerizas J.M., Torres-Padilla M.E. (2015). Early embryonic-like cells are induced by downregulating replication-dependent chromatin assembly. Nat. Struct. Mol. Biol..

[53] Pera M.F., de Wert G., Dondorp W., Lovell-Badge R., Mummery C.L., Munsie M., Tam P.P. (2015). What if stem cells turn into embryos in a dish?. Nat. Methods.

